# Linking Morphological and Genetic Differentiation Among Populations in Seagrass Species With Contrasting Dispersal Strategies

**DOI:** 10.1002/ece3.74571

**Published:** 2026-11-03

**Authors:** Laura K. Reynolds, Alexandra L. Bijak, Savanna C. Barry, Thomas K. Frazer, Charles A. Jacoby, Christine B. Rohal, Jamila Roth

**Affiliations:** ^1^ Soil, Water, and Ecosystem Sciences Department University of Florida IFAS Gainesville Florida USA; ^2^ Nature Coast Biological Station University of Florida IFAS Gainesville Florida USA; ^3^ College of Marine Science, University of South Florida St. Petersburg Florida USA

## Abstract

Long‐distance dispersal sustains genetic diversity across populations and facilitates recovery from disturbance, and dispersal also may shape how ecologically important morphological traits shift in response to environmental change. We examined links among morphology, genetic differentiation, and dispersal through surveys of traits and genetics for *Thalassia testudinum* and *Halodule wrightii* distributed across a naturally occurring gradient in concentrations of total phosphorus in the water column along Florida's Gulf Coast (USA). We expected the contrasting dispersal strategies of the two seagrasses would drive patterns in genetic differentiation: *T. testudinum* produces floating fruits that can disperse over longer distances than the negatively buoyant seeds of *H. wrightii* . Genetic differentiation among populations was higher for *H. wrightii* (*G'*_ST_ = 0.02–0.15) than for *T. testudinum* (*G'*_ST_ = 0.00–0.07), supporting the expectation that limited dispersal leads to more genetic structure. Morphology for both species varied along the phosphorus gradient, where leaves were generally shorter in the estuarine system with the lowest phosphorus concentrations, with more pronounced differences in leaf length observed for *T. testudinum* . The role of the environment in driving leaf length was tested in a common garden experiment with plants of both species collected from three estuarine systems along the phosphorus gradient. Initially, leaf lengths of both species differed across plants originating from different systems. Leaf lengths became more similar throughout the experiment, with leaves of *H. wrightii* from the northernmost system beginning to lengthen after 11 months. Together, our results suggest that responses to environmental change can differ across seagrass species, where the species capable of long‐distance dispersal exhibited greater variability in a morphological trait along an environmental gradient despite less genetic differentiation than the species with more limited dispersal.

## Graphical abstract

We surveyed leaf length and genetic variation in two seagrass species with contrasting life histories across a natural environmental gradient and conducted a common garden experiment to assess variability in an ecologically important trait. Our results demonstrate that responses to environmental change can differ across seagrass species, in which the species capable of long‐distance dispersal exhibited greater variability in a morphological trait along the environmental gradient despite less genetic differentiation than the species with more limited dispersal.

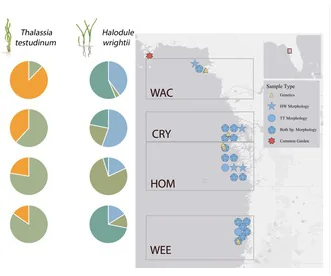

## 1 Introduction

Understanding patterns in genetic and trait differentiation between contrasting dispersal strategies is important for the management and restoration of foundation species in a changing climate. Environmental change can negatively affect foundation species and diminish the ecosystem functions and services they provide (Ansley et al. 2023; Bryan‐Brown et al. 2020; Dunic et al. 2021; Ellison et al. 2005; Krumhansl et al. 2016; Murray et al. 2022; Wernberg et al. 2024). However, species have several mechanisms to respond to environmental change (Reed et al. 2011). Plastic individuals persist through disturbance and stress by altering ecologically relevant traits over relatively short time scales (Jump and Peñuelas 2005; Reed et al. 2011). Plasticity may be advantageous for species that disperse over long distances given they are more likely to encounter heterogeneous environments (Brown 1990; Sultan 2000; Via and Lande 1985). In contrast, plasticity may be less advantageous for species with low dispersal distance, with less plasticity and less genetic exchange among populations favoring local adaptation over long time scales (Angert and Schemske 2005; Clausen et al. 1948; McKay and Latta 2002). Assessing links between genetic differentiation and trait variability across divergent dispersal strategies is a first step in evaluating coastal foundation species' capacities to respond to environmental change.

Seagrasses are coastal foundation species that provide habitat for fish and invertebrates (Orth et al. 1984), stabilize sediments (de Boer 2007), store carbon (Duarte et al. 2013), and reduce concentrations of pollutants and pathogens in the water column (Lamb et al. 2017; Moore 2004). These submerged marine plants are adapted to live in dynamic coastal and estuarine environments, where they respond to local variability in light, temperature, salinity, and availability of nutrients (Lee et al. 2025, 2007; Touchette 2007). Seagrasses comprise ~72 species with a wide spectrum of life history traits (Kilminster et al. 2015; Short et al. 2011) that enable them to persist under highly variable conditions in coastal environments through phenotypic plasticity or dispersal to more favorable environments (Pazzaglia et al. 2021). Species can have distinct dispersal strategies, producing propagules—fruits and seeds—with varying levels of buoyancy, dormancy, and time to maturation (Kendrick et al. 2012). The spatial distribution of genetic diversity within and among seagrass meadows (i.e., population genetic structure) can provide indirect evidence of dispersal strategies. For example, the spatial scale at which genetic distance becomes correlated with geographic distance may suggest the effective limit of a species' dispersal (Kendrick et al. 2012). For seagrasses, alignment between dispersal, population structure, and trait differentiation has not been well studied.

*Thalassia testudinum* and *Halodule wrightii* offer an opportunity to expand our understanding of relationships among dispersal, population structure, and trait variability because they are seagrasses with contrasting dispersal strategies that often co‐exist in mixed meadows in the tropical and subtropical Atlantic. As a climax species, *T. testudinum* has a long life span, low shoot turnover, and high resistance to disturbance, while *H. wrightii* is a short‐lived, pioneering species with high shoot turnover and low resistance to disturbance (Kilminster et al. 2015). *Thalassia testudinum* produces fruits that are positively buoyant, leading to dispersal over long distances (100–1000 km: van Dijk et al. 2009), though the seeds disperse over much shorter distances (< 1–10 m) and do not create a seed bank because they lack a hard seed coat and do not undergo dormancy (Orpurt and Boral 1964; van Tussenbroek et al. 2006). Conversely, *H. wrightii* releases fruits at or beneath the sediment surface, which limits dispersal away from the parent plant (van Tussenbroek et al. 2010). *Halodule wrightii* seeds can remain dormant and viable in a seed bank at densities > 5000 seeds m^−2^ (Darnell et al. 2021) for at least 4 years (McMillan 1991). Past studies have found evidence of weak genetic structure among populations of *T. testudinum* (Bricker et al. 2011; van Dijk et al. 2009) and varying levels among populations of *H. wrightii* (Digiantonio et al. 2020; Larkin et al. 2017). These results suggest greater dispersal for *T. testudinum* ; however, existing data do not provide a rigorous comparison because studies of each species were *c*onducted at different locations and spatial scales. The same is true for past work on trait variation, with both species exhibiting some degree of morphological plasticity when studied at different locations (McDonald et al. 2016; Vivanco Bercovich et al. 2019). Furthermore, most studies investigating genetic or trait differentiation also focus on only one species rather than comparing patterns across multiple, co‐occurring species.

Environmental gradients are natural laboratories for studying drivers of genetic and trait variation. Both *T. testudinum* and *H. wrightii* grow along a naturally occurring gradient in total phosphorus concentrations in the waters along the Gulf coast of peninsular Florida (USA). Past work has shown that the morphology of *T. testudinum* changes along this gradient (Barry et al. 2017), though there have not been any comparisons of morphology for another species common along the gradient, *H. wrightii* —a seagrass with a very different dispersal strategy. While researchers have demonstrated that seagrasses can respond to concentrations of nutrients (Fourqurean et al. 1995; Jensen and Bell 2001; Thomsen et al. 2022), fewer studies have specifically investigated the interaction between morphological responses to the direct and indirect effects of nutrient enrichment and underlying genetic variation.

In this study, we compared genetic structure and morphological variation in *T. testudinum* and *H. wrightii* by measuring plant morphology and genetic differentiation among populations along a gradient of total phosphorus concentrations in the water column. We further assessed the role of the environment in driving morphological differences in these species by conducting a common garden experiment. We assessed responses for both species by measuring variation in leaf length among plants collected from different estuarine systems along the phosphorus gradient coupled with analyses of population genetics and by measuring the relative changes in leaf length between populations grown in the common garden experiment. High variability without genetic differentiation suggests higher environmental control, and persistence of differences in leaf lengths during the common garden implies lower environmental control. Based on their contrasting dispersal strategies, we hypothesized *T. testudinum* would exhibit less genetic differentiation than *H. wrightii* . We expected a similar trend in morphological variation along the phosphorus gradient for both species but with the environment having a stronger influence on trait variation in *T. testudinum* , as evidenced by limited genetic differentiation across populations and converging morphologies for plants collected from different populations in the common garden experiment.

## 2 Methods

### 2.1 Systems

This study was conducted along a persistent, north‐to‐south decrease in concentrations of total phosphorus in the waters along the Gulf coast of peninsular Florida spanning four coastal riverine systems: Waccasassa, Crystal, Homosassa, and Weeki Wachee (Figure 1; Barry et al. 2024; Barry et al. 2017; Frazer et al. 1998; Jacoby and Frazer 2015; Jacoby and Frazer 2013). Past work also has documented changes in the morphology of *T. testudinum* along this gradient: leaf height and area decrease with decreasing concentrations of total phosphorus, and leaves are especially small at sites furthest south where concentrations of phosphorus are lowest (Table 1; Barry et al. 2017). Sampling for all components of this study was conducted in shallow (1–3 m depth), subtidal seagrass meadows within the four systems; however, the exact location of sample sites for each component differed due to interannual variability in seagrass cover (Figure 1).

**FIGURE 1:**
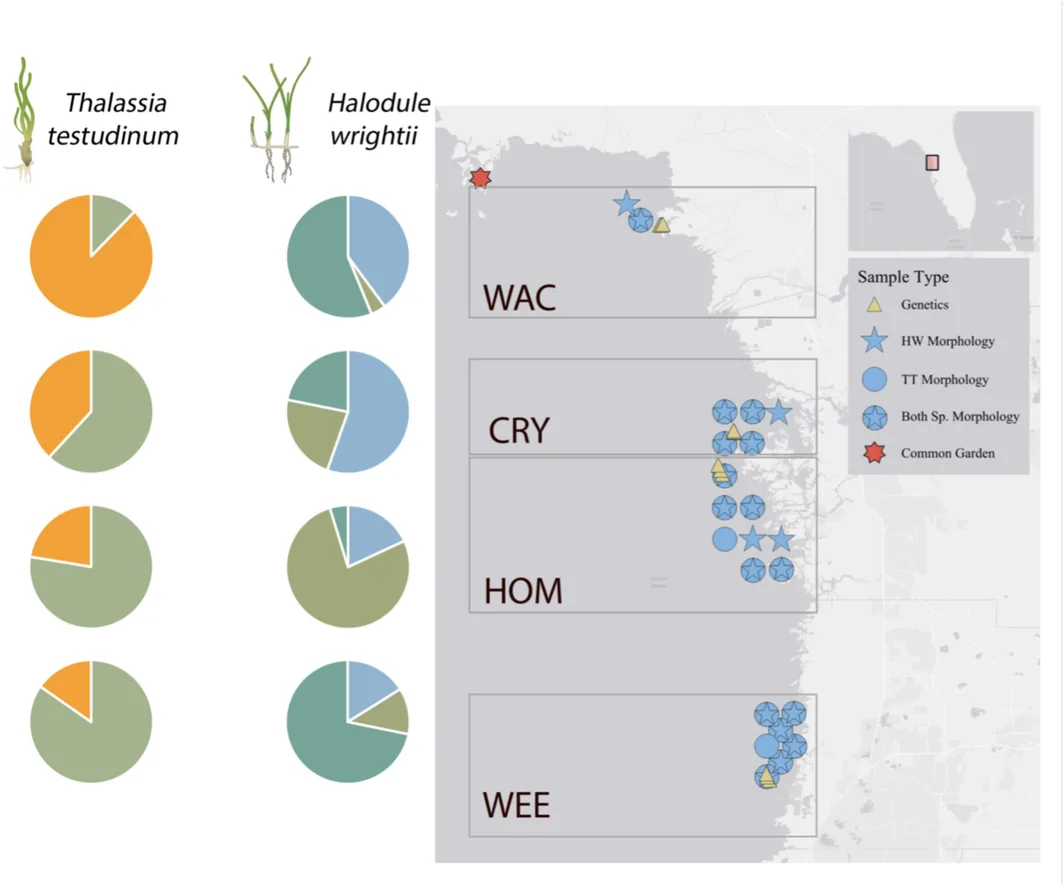
Map displaying sites where samples for morphological and genetic analyses were collected and the location of the common garden experiment (CRY = Crystal, HOM = Homosassa, WAC = Waccasassa, WEE = Weeki Wachee). Genetic cluster membership for *Thalassia testudinum* (TT; left) and *Halodule wrightii* (HW; right) at each system based on ancestry coefficients from “tess3r” analysis, where *K* = 2 for *T. testudinum* and *K* = 3 for *H. wrightii* . Pie charts represent average cluster assignment values across all individuals within a system and are colored by cluster number: For *T. testudinum* , Cluster 1 = green and Cluster 2 = orange; for *H. wrightii* , Cluster 1 = light blue, Cluster 2 = green, and Cluster 3 = teal.

**TABLE 1:** Water column characteristics for sampling sites along the phosphorus gradient (CRY = Crystal, HOM = Homosassa, WAC = Waccasassa, WEE = Weeki Wachee) and for Cedar Key seawater supplying the common garden (CG).

Site	Depth (m)	Temp (°C)	Salinity (ppt)	DO (mg L^−1^)	TN (μg L^−1^)	TP (μg L^−1^)	*I*_*z*_ (μE m^−2^ s^−1^)
CG	n/a	23.2 ± 4.6	27.5 ± 1.3	7.5 ± 0.8	580.8 ± 146.0	62.4 ± 17.8	640.3 ± 477.9
WAC	1.2 ± 0.4	22.6 ± 6.1	24.4 ± 4.8	7.2 ± 1.8	486.4 ± 166.6	25.8 ± 17.0	497.9 ± 296.8
CRY	1.0 ± 0.4	22.9 ± 6.1	23.3 ± 3.7	7.2 ± 1.9	367.1 ± 135.4	10.7 ± 3.9	679.3 ± 327.1
HOM	1.3 ± 0.3	23.0 ± 6.0	27.3 ± 4.1	7.0 ± 1.8	368.0 ± 115.8	9.1 ± 3.9	565.6 ± 303.6
WEE	1.1 ± 0.3	23.5 ± 6.0	21.9 ± 4.0	7.1 ± 1.4	441.5 ± 133.8	7.1 ± 2.6	776.6 ± 330.3

*Note:* Mean and standard deviation are provided for water depth, temperature (Temp), salinity, dissolved oxygen (DO), total nitrogen (TN), total phosphorus (TP), and irradiance at mean bottom depth (*I*_*z*_). Data for the sampling sites represent long‐term averages (measured monthly 1999–2012) from Barry et al. (2017). Nutrient concentrations for the seawater supplied to the common garden are averages from monthly measurements collected over the course of the experiment (Florida LAKEWATCH 2021) while temperature and irradiance data are from point measurements reported in Bijak et al. (2025).

### 2.2 Morphology

We analyzed existing data from a sampling program that collected seagrass leaf lengths from 2010 to 2012 to test whether the lengths of *T. testudinum* and *H. wrightii* leaves varied across each of the four systems along the phosphorus gradient. Each year, leaves were collected from up to seven sites per system in May–June (early) or August–September (late), or both periods (Figure 1). In the laboratory, lengths were measured from the base of the meristem to the top of the longest intact leaf. Sampling was unequal across systems and years because the abundance of both species varied in space and through time (for both species, *n* = 0–7 sites sampled within each system each year).

To accommodate an unbalanced design, we assessed spatiotemporal differences in leaf lengths separately for each species using linear mixed models (“lme4” package; Bates et al. 2015) in R version 4.5.1 (R Core Team 2025). System, year, and their interaction were included as fixed effects, with season included as an additional fixed effect to account for variation across the early versus late part of the growing season. Replicate quadrats (≤ 10) sampled within quadrants (≤ 4) at each site were included as random effects to account for the non‐independence of multiple leaves measured within the same site. Model assumptions were evaluated by examining quantile–quantile plots of model residuals and plots of residuals versus fitted values. Homogeneity of residual variance among systems was assessed by comparing residual distributions among systems, and where appropriate, heterogeneous residual variances among systems were modeled using a variance structure that allowed system‐specific residual variances using the “nlme” package (Pinheiro and Bates 2025, 2000).

Significance of fixed effects was assessed using analysis of variance (ANOVA). When significant effects or interactions were detected, estimated marginal means were calculated from the fitted models to estimate mean leaf length for each system for each year while accounting for the other terms in the model. Pairwise comparisons among estimated marginal means were conducted using Šídák‐adjusted comparisons, with *α* = 0.05 considered statistically significant.

### 2.3 Common Garden Experiment

We collected *H. wrightii* and *T. testudinum* ramets (individual plants) from each of three systems along the phosphorus gradient—Crystal, Homosassa, and Weeki Wachee—in July 2021. The common garden experiment was conducted in an open, flow‐through system plumbed with seawater in Cedar Key, a site north of the documented phosphorus gradient that is more balanced in terms of nitrogen and phosphorus concentrations (Table 1; Figure 1). All ramets included vertical and horizontal rhizomes, with ramets of *T. testudinum* containing a single shoot and ramets of *H. wrightii* containing three connected shoots. Individual ramets were planted in separate plastic pots (15.2‐cm diameter) filled with a 3:1 mixture of sterile sand and organic marsh mud, and pots were placed in 378.5‐L tanks. Three replicate pots for each combination of species and system were distributed among six tanks (*n* = 18).

We measured leaves approximately every week for ~11 months (August 2021–June 2022). For *T. testudinum* , the length of the primary leaf was measured, and for *H. wrightii* , the lengths of the longest leaves in each of the three connected shoots were measured and averaged for a single value per pot. Lengths were measured from the sediment surface to the top of the leaf and were functionally similar to morphological measurements from the field surveys. Different leaves were measured over the course of the experiment, given plastochron intervals in Gulf of Mexico populations range from 10.6–26.1 and 8–16.5 days for *T. testudinum* (Duarte 1991; Gallegos et al. 1992; van Tussenbroek 2002) and *H. wrightii* (Gallegos et al. 1994; Hauxwell et al. 2001; Short and Duarte 2001), respectively.

For each species, we assessed differences in leaf lengths through time using a mixed model ANOVA implemented with “lme4” and “lmerTest” in R (Bates et al. 2015; Kuznetsova et al. 2017), with pot nested within tank as random effects and system and sampling date (coded as a numeric variable) as independent and interactive fixed effects. An AR(1) covariance structure was added to account for repeated measures. Leaf lengths for *T. testudinum* and *H. wrightii* were BoxCox and log‐transformed, respectively, to meet the assumptions of normality and homogeneity of variance, as confirmed by graphically evaluating residuals. We conducted Kruskal–Wallis rank sum tests to evaluate differences in the lengths of leaves among systems at the start and end of the experiment for each species. Non‐parametric tests were chosen due to unequal sample sizes and non‐homogeneous variances that emerged at the end of the experiment due to plant mortality. Dunn's multiple comparison tests with Bonferroni correction were conducted for post hoc pairwise comparisons with the “dunn.test” package (Dinno 2026). Additionally, we conducted Wilcoxon rank‐sum tests for all pairwise comparisons to calculate effect sizes and estimate median differences with 95% confidence intervals. Effect sizes were quantified using rank‐biserial correlation (*r*).

### 2.4 Genetic Analysis

In July 2019, we collected leaves of both *T. testudinum* and *H. wrightii* at 1–3 sites within each of the four systems along the gradient in phosphorus concentrations (Figure 1). At each site, leaves were collected haphazardly from shoots at least 5 m apart. The total number of samples collected per site differed ( *T. testudinum* : *n* = 47–49; *H. wrightii* : *n* = 16–36) because the shoot density and extent of seagrass cover for each species varied within and across systems. Leaves were stored at −80°C until analysis.

Leaves were homogenized and DNA was extracted using Qiagen DNeasy Plant Mini kits per manufacturer's instructions. We amplified DNA at eight species‐specific microsatellite loci using established PCR conditions for both *T. testudinum* (Van Dijk et al. 2007) and *H. wrightii* (Larkin et al. 2012). The PCR products were analyzed on an Applied Biosystems 3730 DNA analyzer at the Georgia Genomics and Bioinformatics Core (University of Georgia), and fragment lengths were determined using Geneious v11.1.2 (https://www.geneious.com) with the microsatellite plugin.

For each species from each system (data were pooled across sites), we estimated the probability that replicates of a clonal lineage were derived from separate sexual events (*p*_SEX_) using the software GENCLONE 2.0 (Arnaud‐Haond and Belkhir 2007). We identified clonal lineages as those with a *p*_SEX_ < 0.01 to avoid misidentifying lineages created by scoring errors or somatic mutations (Arnaud‐Haond et al. 2007; Arnaud‐Haond and Belkhir 2007). For each species from each system, we calculated diversity metrics including clonal richness as (*G* − 1)/(*N* − 1), where *G* is the number of clonal lineages and *N* is the number of samples, along with the number of alleles, expected heterozygosity, and inbreeding coefficients in GenAlEx 6.5 (Peakall and Smouse 2012, 2006). We examined genetic differentiation among systems via pairwise *G'*_ST_ (Hedrick 2005) calculated in Genodive 3.06 (Meirmans 2020; Meirmans and Van Tienderen 2004) and analyzed differences in pairwise *G'*_ST_ among species using paired Wilcoxon signed‐rank tests (“stats” package in R).

We examined how genetic differentiation varied with geographic distance by testing for isolation by distance: we calculated the correlation between geodesic (“gmt” package) and Nei's genetic (“adegenet” package) distances using a Mantel's test (“ade4” package) in R (Dray and Dufour 2007; Jombart and Ahmed 2011; Magnusson 2022). We further assessed population structure using two Bayesian clustering methods. First, we estimated ancestry coefficients independent of spatial patterns in STRUCTURE v2.2.3 (Pritchard et al. 2000) and determined the most likely number of genetic clusters, *K*, via the Δ*K* method (Evanno et al. 2005). We then estimated ancestry coefficients while accounting for spatial patterns and determined *K* using cross‐entropy methods in the “tess3R” package in R (Caye et al. 2016).

## 3 Results

### 3.1 Morphology

The morphology of both *T. testudinum* and *H. wrightii* varied along the phosphorus gradient (Figure 2), with leaves being shortest in the southernmost system, Weeki Wachee ( *T. testudinum* = 161.9 ± 1.5 mm; *H. wrightii* = 115.4 ± 1.4 mm; mean ± standard error, here and subsequently), and longest in the northernmost systems, Waccasassa ( *T. testudinum* = 577.9 ± 15.0 mm) and Crystal ( *H. wrightii* = 311.2 ± 7.2 mm). For both species, leaf lengths differed across systems and within systems across years (Table A1; Figure 2). Leaves generally decreased in length from the northernmost to the southernmost system, where we identified the strongest evidence of consistent differences between Waccasassa and Weeki Wachee in *T. testudinum* . Leaf lengths for *H. wrightii* were significantly different between Crystal and Weeki Wachee but only in some years. Across all systems and years, *T. testudinum* exhibited greater morphological variability than *H. wrightii*, with overall coefficients of variation being 62.1% and 39.0%, respectively.

**FIGURE 2:**
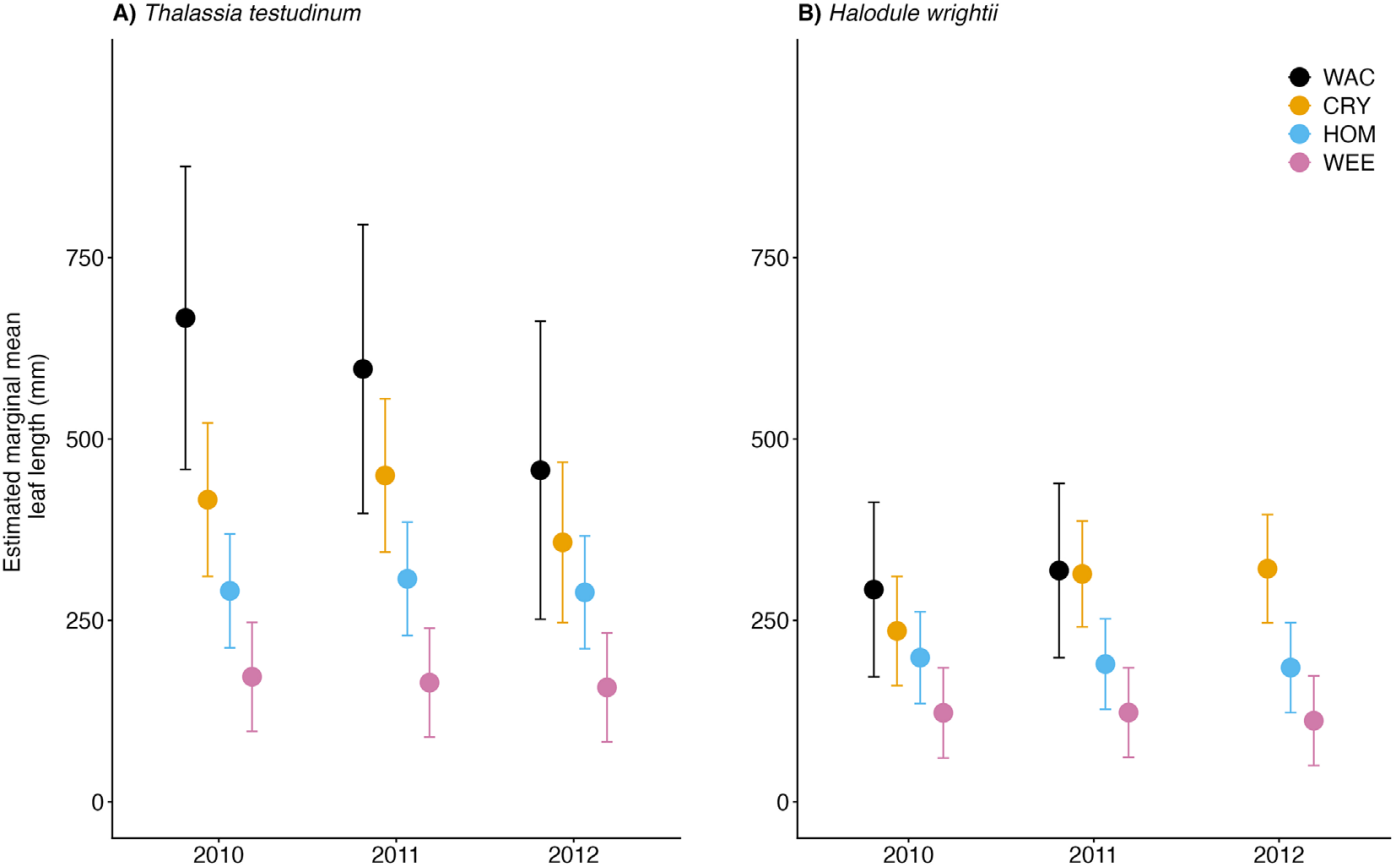
Estimated marginal mean leaf lengths for (A) *Thalassia testudinum* and (B) *Halodule wrightii* for each system along the phosphorus gradient in 2010–2012. Systems are denoted by color: Crystal (CRY = yellow); Homosassa (HOM = blue); Weeki Wachee (WEE = maroon). *Halodule wrightii* was absent from Waccasassa in 2012.

### 3.2 Common Garden Experiment

Leaf lengths for *T. testudinum* and *H. wrightii* varied among systems over the duration of the common garden experiment as indicated by the system × date interactions (Table A2; Figure 3). Leaf lengths for both species differed among systems at the beginning of the experiment ( *T. testudinum* *χ* ^2^ = 28.54, *p*‐value = 6.33e‐07; *H. wrightii* *χ* ^2^ = 27.05, *p*‐value = 1.33e‐06): leaves from Homosassa and Crystal were initially longer than leaves from Weeki Wachee (Tables A2–A4). At the experiment midpoint, leaf lengths were similar across systems for both species. By the end of the experiment, the overall difference in *H. wrightii* leaf lengths among populations was marginally nonsignificant (Kruskal–Wallis: *χ* ^2^ = 5.41, df = 2, *p*‐value = 0.067); however, pairwise comparisons revealed that leaves from Crystal were longer than leaves from Weeki Wachee (Table A3; median difference = 67.1 mm, rank‐biserial *r* = 0.74, *p*‐value = 0.042). In contrast, *T. testudinum* leaves for plants collected from different systems remained similar in length at the end of the experiment (*χ* ^2^ = 2.71, *p*‐value = 0.259; Table A4).

**FIGURE 3:**
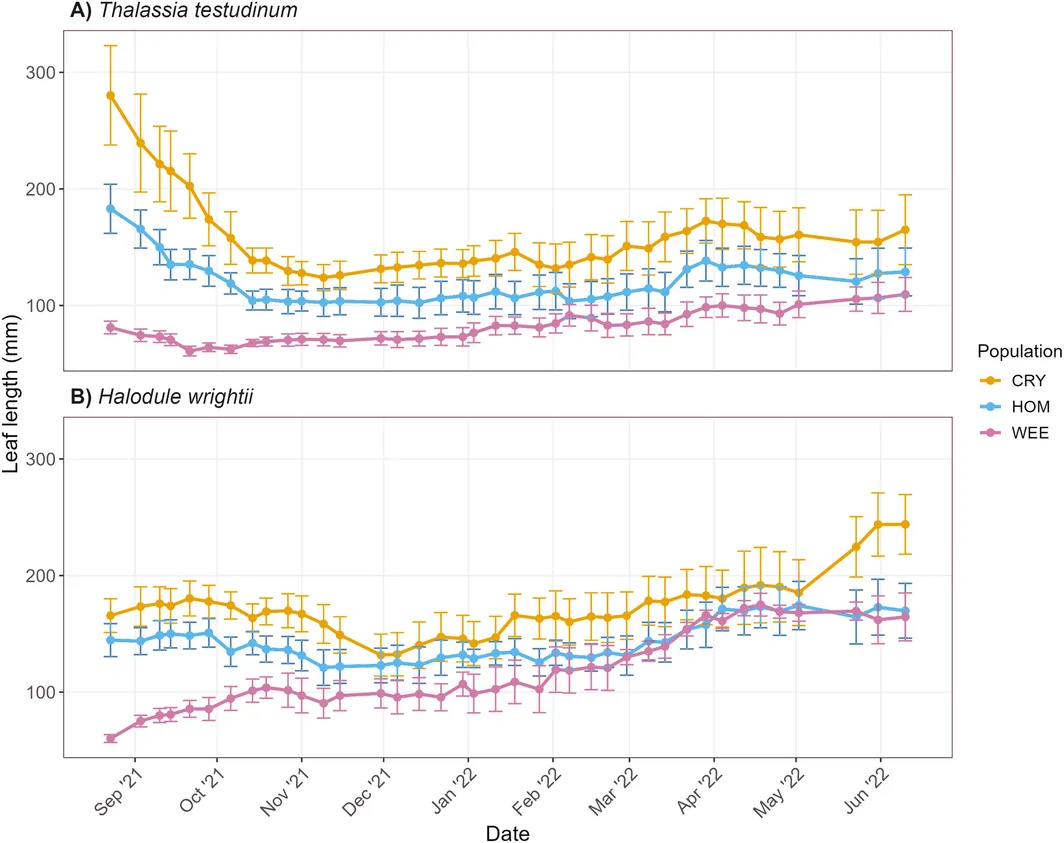
Mean leaf lengths ± standard errors for (A) *Thalassia testudinum* and (B) *Halodule wrightii* growing in a common garden from August 2021 to June 2022. Systems are denoted by color: Crystal (CRY = yellow); Homosassa (HOM = blue); Weeki Wachee (WEE = maroon).

### 3.3 Genetic Analysis

*Thalassia testudinum* and *H. wrightii* exhibited similar levels of genetic diversity across systems (Figure 4). For both species, clonal richness was lowest in the northernmost system, Waccasassa ( *T. testudinum* : 0.29; *H. wrightii* : 0.20), and highest in the southernmost system, Weeki Wachee ( *T. testudinum* : 0.59; *H. wrightii* : 0.69). Across both species and all systems, the mean number of alleles per locus ranged from 3.13 to 6.00, expected heterozygosity ranged from 0.54 to 0.63, and the inbreeding coefficient varied between −0.19 and 0.16. Negative inbreeding coefficients—indicating heterozygote excess—were more frequently observed in *H. wrightii* .

**FIGURE 4:**
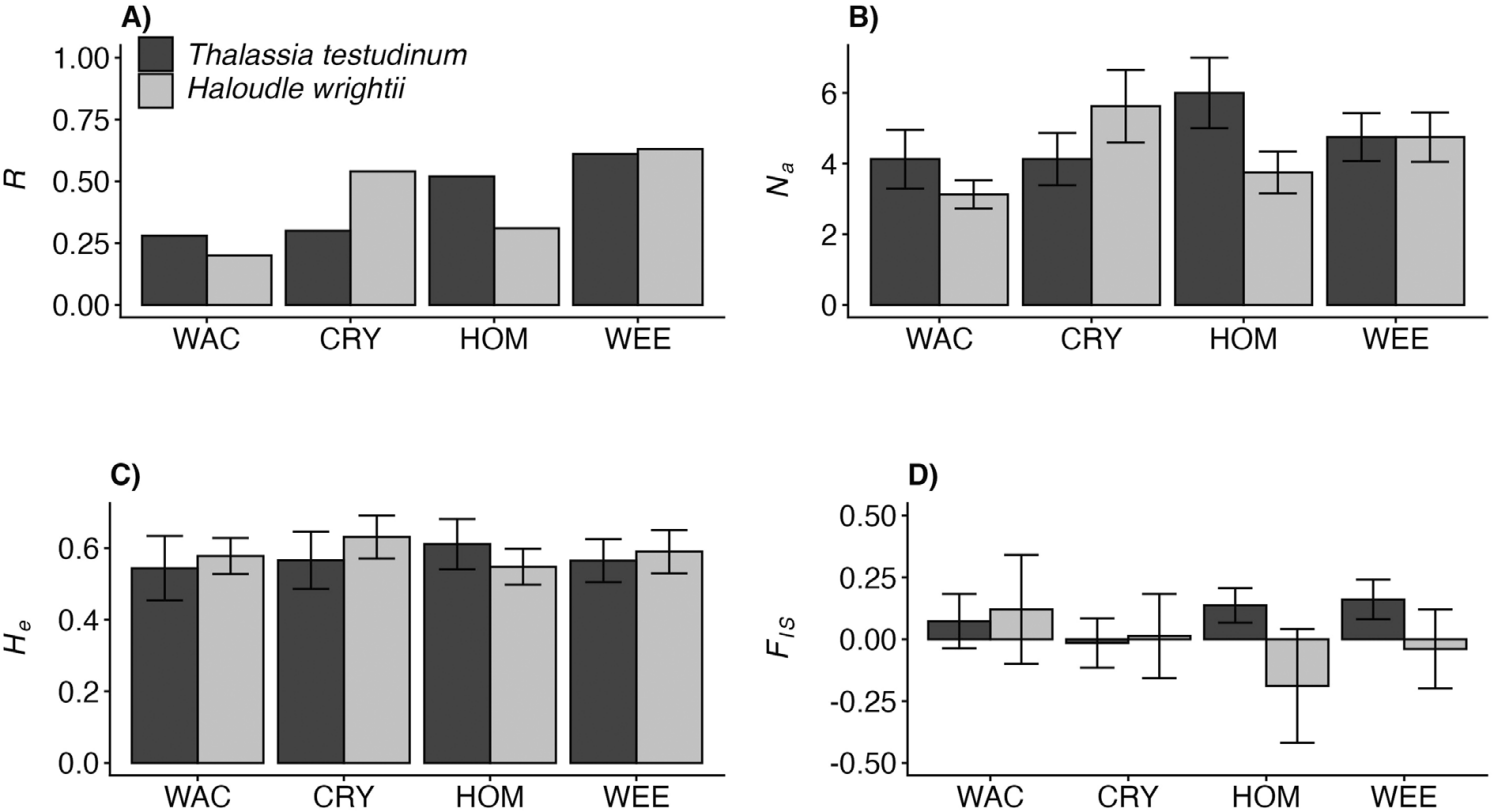
Mean genetic diversity metrics across all microsatellite loci by system (CRY = Crystal, HOM = Homosassa, WAC = Waccasassa, WEE = Weeki Wachee) for each species (black = *Thalassia testudinum* , gray = *Halodule wrightii* ): (A) clonal richness, *R*; (B) number of alleles, *N*_A_; (C) expected heterozygosity, *H*_e_; and (D) inbreeding coefficient, *F*_IS_. Error bars are standard errors.

There was evidence of greater genetic differentiation among systems in *H. wrightii* . Across most pairs of systems (except Waccasassa‐Weeki Wachee and Wacasassa‐Crystal), *G'*_ST_ values were higher for *H. wrightii* than for *T. testudinum* (Figure 5); however, there was no evidence of a statistically significant difference in *G'*_ST_ between species (*Z* = −1.53, *p*‐value = 0.125). Genetic differentiation was related to geographic distance for *H. wrightii* as determined by isolation by distance (*r* = 0.73, *p*‐value = 0.002) but not for *T. testudinum* (*r* = 0.10, *p*‐value = 0.283).

**FIGURE 5:**
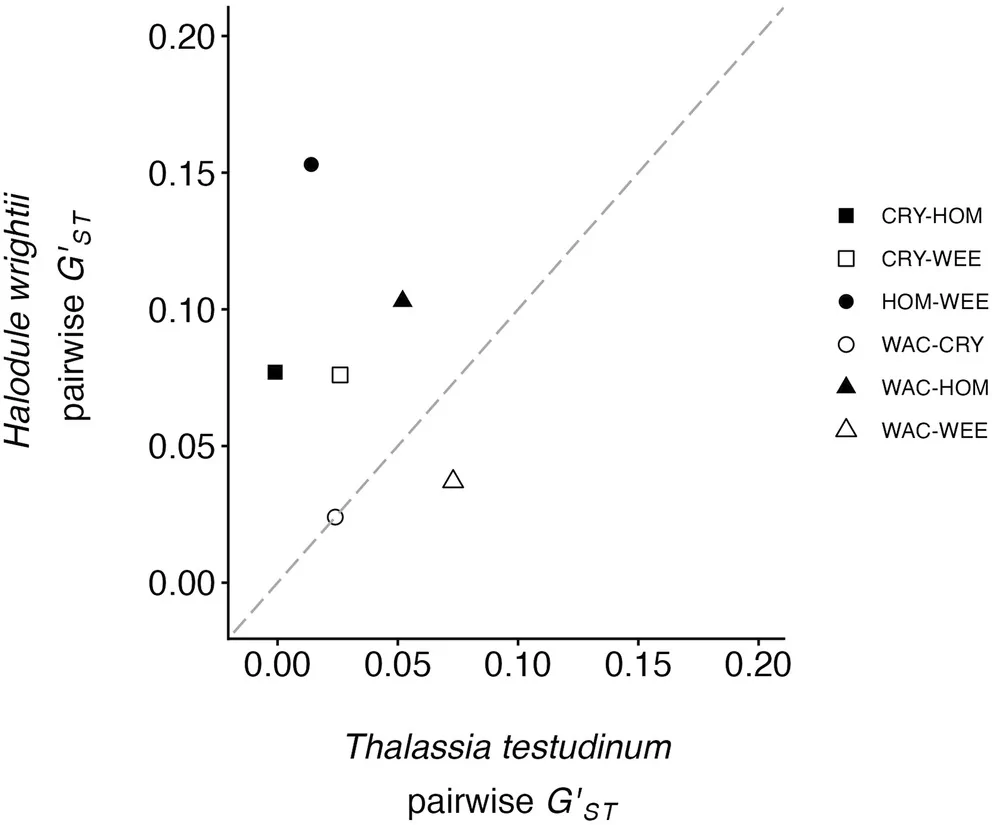
Relationship between pairwise *G'*_ST_ values for *Thalassia testudinum* (*x*‐axis) and *Halodule wrightii* (*y*‐axis), with a 1:1 line included for reference.

We found evidence for population structure across the study area for both species (Figure 1). We identified two distinct genetic clusters of *T. testudinum* and at least three distinct genetic clusters of *H. wrightii* , though there may be finer levels of genetic structure for *H. wrightii* . Results were similar for both approaches to assigning populations, though the spatially independent approach (i.e., STRUCTURE) suggested four rather than three clusters for *H. wrightii* (Figures A1 and A2). For *T. testudinum* , cluster membership was similar across the three southernmost systems where Cluster 1 was dominant, while Cluster 2 was dominant in Waccasassa. For *H. wrightii* , Cluster 1 was more prevalent in the two northern systems (Waccasassa and Crystal), Cluster 2 dominated Homosassa, and Cluster 3 dominated the southernmost system (Weeki Wachee).

## 4 Discussion

Seagrasses with contrasting dispersal strategies can respond similarly to environmental variation. In our study, leaf length for both *T. testudinum* and *H. wrightii* generally increased along a gradient of increasing phosphorus concentrations in the water column, though the two species showed different levels of morphological variability and genetic differentiation along the gradient. *Thalassia testudinum* exhibited > 60% more variability in leaf length compared to *H. wrightii* , yet *T. testudinum* meadows were less differentiated and had weaker population structure than *H. wrightii* meadows, in line with expectations for populations of species with high versus limited dispersal capacities. When both species were transplanted into a common garden, relative differences in leaf length among systems diminished for both species and began to re‐emerge for *H. wrightii* . Together, these findings suggest that seagrass species occupying overlapping ecological niches may respond similarly to environmental variability yet differ in the magnitude and variance of those responses—a divergence that may, in part, reflect distinct dispersal strategies. The underlying mechanisms generating variable trait responses across environmental gradients warrant further attention, given their important implications for seagrass management, restoration, and conservation.

Despite varying levels of genetic differentiation across systems, both species responded to the environment similarly in that leaf length varied across the phosphorus gradient. Leaf lengths were shortest in Weeki Wachee, the southernmost system where total phosphorus concentrations in the water column are lowest, and highest in more northern systems where phosphorus concentrations were higher (Figure 2). Differences in morphology were present in the common garden experiment initially, but leaf lengths later converged for both species (Figure 3). Some differences in morphology were present by the end of the experiment for *H. wrightii* , but not for *T. testudinum* (Figure 3). We expected leaf lengths for both species to decrease initially due to transplant stress (Govers et al. 2015; Horn et al. 2009; Park and Lee 2007) as well as seasonal changes in biomass, as both species in the northern Gulf of Mexico experience declining biomass in the fall and winter (often September through March; Dawes 1985; Dawes et al. 1985; Iverson and Bittaker 1986; Kowalski et al. 2001). Relative differences in leaf lengths between plants collected from the most distant systems, Crystal and Weeki Wachee, were observed at the beginning and end of the common garden experiment in *H. wrightii* but not in *T. testudinum* (Figure 3). While it is possible that a longer common garden experiment conducted across more than a single growing season may have led to the re‐emergence of more differences in both species, stronger genetic differentiation and lower trait variability among populations supports the hypothesis that as a species with limited dispersal, *H. wrightii* exhibits less plastic responses to changes in nutrient concentrations.

Differentiation based on neutral genetic markers does not necessarily reflect processes related to natural selection or adaptation (Kirk and Freeland 2011), and there are also non‐hereditary genetic mechanisms that can lead to genetic and morphological differences between populations in seagrasses. For example, accumulated somatic mutations can lead to population differentiation via somatic genetic drift (Yu et al. 2020). Further epigenetic changes such as DNA methylation, can cause differences in fitness‐related traits, like photosynthetic performance (Jueterbock et al. 2020). Though more work is needed to parse the relative effects of local adaptation versus plasticity on morphological variation in seagrasses, we found that levels of genetic differentiation, and to a lesser extent trait variability, can reflect dispersal strategies.

In contrast to genetic differentiation, the levels of genetic diversity levels we found in *T. testudinum* and *H. wrightii* do not completely align with our understanding of contrasting dispersal strategies between the two species. Both species had similar levels of genetic diversity, though we would have expected higher genetic diversity in *T. testudinum* than we observed, given the greater potential for long‐distance dispersal in this species (van Dijk et al. 2009). Genotypic richness (*R* = 0.43 ± 0.08; Figure 4A) and the number of alleles (*N*_a_ = 4.75 ± 0.44; Figure 4B) were low for *T. testudinum* along Florida's Gulf Coast relative to other populations in Florida Bay (Bricker et al. 2011) and throughout the Caribbean (van Dijk et al. 2018), though differences in sample size (e.g., higher sample size increases the probability of detecting rare alleles) and/or the spatial scale of sampling (e.g., samples collected at shorter distances are more likely to be clones) across studies prevent the direct comparison of these metrics (Arnaud‐Haond and Belkhir 2007). Genotypic richness (*R* = 0.42 ± 0.10; Figure 4A) and the number of alleles (*N*_a_ = 4.31 ± 0.55; Figure 1B) in *H. wrightii* were similar to those observed in other populations in Florida and in Texas coastal lagoons (Larkin et al. 2017; Reynolds et al. 2019). In both species, overall mean expected heterozygosity was moderately high ( *T. testudinum* *H*_e_ = 0.57 ± 0.01; *H. wrightii* *H*_e_ = 0.59 ± 0.02; Figure 4C) and inbreeding coefficients were close to zero ( *T. testudinum* *F*_IS_ = 0.09 ± 0.04; *H. wrightii* *F*_IS_ = −0.02 ± 0.06; Figure 4D). Moderately high genotypic richness and genetic diversity in *H. wrightii* relative to other Gulf of Mexico populations, despite evidence of restricted gene flow, suggests mechanisms other than sexual reproduction and subsequent propagule dispersal (i.e., somatic mutation and epigenetic changes) may be contributing. In addition, genetic mosaicism occurs in *H. wrightii* , where an individual plant can contain cells with different genotypes, which can elevate diversity metrics (da Silva et al. 2017; Digiantonio et al. 2020).

The species‐specific patterns in genetic and morphological variation we found could influence recovery from disturbances and potential for local adaptation and should therefore be considered in the management, restoration, and conservation of seagrasses. If further work confirms high trait variability is a plastic response to the environment, restoration practitioners can be flexible when selecting source populations because *T. testudinum* may be able to quickly adapt to different conditions. High dispersal and weak population structure in *T. testudinum* also limit the risk of displacing locally adapted genotypes. Practitioners may have less flexibility in selecting sources for *H. wrightii* because of its comparably lower dispersal, less evidence of population structure, and less trait variability. These differences also have implications for natural recovery following disturbance and adaptation to climate change. *Halodule wrightii* may be more resilient and may recover faster from extreme events, such as storms (i.e., more resilient Kilminster et al. 2015; Williams 1990), but this species may not be as adept at populating new locations if its dispersal is limited (Kendrick et al. 2016, 2012). *Thalassia testudinum* has greater dispersal capacity and is more resistant to extreme events than *H. wrightii* , but it grows slower and does not recover from disturbance as quickly (i.e., less resilient; Kilminster et al. 2015; Roth et al. 2024), so it is especially important to protect existing populations to ensure long‐term persistence of this species.

Our findings highlight that dispersal strategy can predict broad patterns in genetic differentiation and morphological variation along an environmental gradient for two co‐existing seagrass species, *T. testudinum* and *H. wrightii* , in the subtropical Atlantic. However, more evidence from common garden and/or transplant experiments that test provenance effects on plant traits is needed to evaluate the mechanisms underlying trait variability in natural environments, including phenotypic plasticity and local adaptation. Both genetic and non‐genetic drivers of trait variation are likely important for long‐term population persistence for seagrasses and should therefore be considered in restoration and adaptation planning. More plastic plants may fare better under greater environmental variability anticipated with climate change; however, genetic variation is also necessary to avoid population bottlenecks and allow for longer‐term evolutionary processes, like local adaptation, to occur.

## Author Contributions

**Laura K. Reynolds:** conceptualization (equal), data curation (equal), formal analysis (equal), investigation (equal), visualization (equal), writing – original draft (equal), writing – review and editing (equal). **Alexandra L. Bijak:** data curation (equal), formal analysis (equal), visualization (equal), writing – original draft (equal), writing – review and editing (equal). **Savanna C. Barry:** conceptualization (equal), data curation (equal), investigation (equal), writing – review and editing (equal). **Thomas K. Frazer:** data curation (equal), investigation (equal), writing – review and editing (equal). **Charles A. Jacoby:** data curation (equal), investigation (equal), writing – review and editing (equal). **Christine B. Rohal:** data curation (equal), formal analysis (equal), investigation (equal), visualization (equal), writing – original draft (equal), writing – review and editing (equal). **Jamila Roth:** data curation (equal), formal analysis (equal), investigation (equal), visualization (equal), writing – original draft (equal), writing – review and editing (equal).

## Funding

This work was supported by the Seagrass Restoration Technology Development Initiative (403.9334 Florida Statutes).

## Conflicts of Interest

The authors declare no conflicts of interest.

## Supporting information


**Table A1:** Results of Type III analysis of variance (ANOVA) for the linear mixed‐effects models evaluating the effects of system, year, system × year interaction, and season on leaf length for both species. The Satterthwaite and containment methods were used to calculate degrees of freedom (DF) for the *H. wrightii* and *T. testudinum* models, respectively.
**Table A2:** Results of Type III analysis of variance (ANOVA) comparing leaf lengths for *Halodule wrigthii* and *Thalassia testudinum* by system of origin, sampling date during the common garden experiment, and their interaction. Kenward–Roger method was used for degrees of freedom (DF) calculation.
**Table A3:** Post hoc pairwise comparisons of *Halodule wrightii* leaf lengths from three systems (CRY: Crystal, HOM: Homosassa, WEE: Weeki Wachee) in the common garden at the initial and final sampling dates. Sample sizes (*n*), group medians, and median differences between sites are shown. Dunn's test with Bonferroni correction was used for multiple comparisons (*Z* = standardized test statistic). Effect sizes were quantified using rank‐biserial correlation (*r*) with 95% confidence intervals (CI) calculated from Wilcoxon rank‐sum tests.
**Table A4:** Post hoc pairwise comparisons of *Thalassia testudinum* leaf lengths from three systems (CRY: Crystal, HOM: Homosassa, WEE: Weeki Wachee) in the common garden at the initial and final sampling dates. Sample sizes (*n*), group medians, and median differences between sites are shown. Dunn's test with Bonferroni correction was used for multiple comparisons (*Z* = standardized test statistic). Effect sizes are quantified using rank‐biserial correlation (*r*) with 95% confidence intervals (CI) calculated from Wilcoxon rank‐sum tests.
**Figure A1:** Delta‐*K* plots from STRUCTURE analyses for (A) *K* = 2–6 for *Halodule wrightii* and (B) *K* = 2–3 for *Thalassia testudinum* .
**Figure A2:** Cross‐entropy plots from tess3r analyses for *K* = 1–8 for (A) *Halodule wrightii* and (B) *Thalassia testudinum* .

## Data Availability

Data from this study are available in the Zenodo repository: https://doi.org/10.5281/zenodo.20692202.
