# Supplementary material for: Linking Morphological and Genetic Differentiation Among Populations in Seagrass Species With Contrasting Dispersal Strategies
Source: Ecol Evol. 2026 Nov 3;16(11):e74571. doi: 10.1002/ece3.74571 (PMC13729265; doi:10.1002/ece3.74571)
Supplement: Supplementary file 1 [file ECE3-16-e74571-s001.pdf]

Appendix

Supplementary information for **Linking morphological and genetic differentiation among populations in seagrass species with contrasting dispersal strategies**

Laura K. Reynolds[1], Alexandra L. Bijak[1], Savanna C. Barry[2], Thomas K. Frazer[3], Charles A. Jacoby[3], Christine B. Rohal[1], Jamila Roth[1]

[1]University of Florida IFAS, Soil, Water, and Ecosystem Sciences Department
[2]University of Florida IFAS, Nature Coast Biological Station
[3]University of South Florida, College of Marine Sciences

Table of Contents

Table A1......2
Table A2......3
Table A3......4
Table A4......5
Figure A1......6
Figure A2......7

Table A1: Results of Type III analysis of variance (ANOVA) for the linear mixed-effects models evaluating the effects of system, year, system-x-year interaction, and season on leaf length for both species. The Satterthwaite and containment methods were used to calculate degrees of freedom (DF) for the *H. wrightii* and *T. testudinum* models, respectively.

Species	Effect	Numerator DF	Denominator DF	*F*	*p*-value
Halodule wrightii	System	3	17.08	6.12	5.07E-03
	Year	2	327.6	5.67	3.81E-03
	System x Year	5	308.01	7.57	1.01E-06
	Season	1	2109.74	30.27	4.21E-08
Thalassia testudinum	System	3	15	12.78	2.07E-04
	Year	2	379	7.06	9.76E-04
	System x Year	6	379	6.4	1.95E-06
	Season	1	2923	43.97	3.95E-11

Table A2: Results of Type III analysis of variance (ANOVA) comparing leaf lengths for *Halodule wrigthii* and *Thalassia testudinum* by system of origin, sampling date during the common garden experiment, and their interaction. Kenward-Roger method was used for degrees of freedom (DF) calculation.

Species	Effect	Numerator DF	Denominator DF	*F*	*p*-value
Halodule wrightii	System	2	1,230.5	115.39	<2.2e-16
	Date	1	1,233.2	54.78	2.49e-13
	System × Date	2	1,231.9	37.69	<2.2e-16
Thalassia testudinum	System	2	1,385.3	151.17	<2.2e-16
	Date	1	1,387.1	0.01	0.93
	System × Date	2	1,386.7	34.99	1.49e-15

Table A3: Post-hoc pairwise comparisons of *Halodule wrightii* leaf lengths from three systems (CRY: Crystal, HOM: Homosassa, WEE: Weeki Wachee) in the common garden at the initial and final sampling dates. Sample sizes (n), group medians, and median differences between sites are shown. Dunn's test with Bonferroni correction was used for multiple comparisons (Z = standardized test statistic). Effect sizes were quantified using rank-biserial correlation (r) with 95% confidence intervals (CI) calculated from Wilcoxon rank-sum tests.

Sampling date	System pairs (1 vs 2)	n_1	n_2	$Median_1$ (mm)	$Median_2$ (mm)	Median Difference (mm)	Dunn z	Dunn p-value	r	CI
Initial	CRY-HOM	17	17	175.0	135.0	40.0	0.91	0.54	0.20	[-2.4, 6.3]
	CRY- WEE	17	15	175.0	65.0	110.0	4.92	1.28e-06	1.00	[5.8, 13.8]
	HOM- WEE	17	15	135.0	65.0	70.0	4.03	8.05e-07	0.85	[5.3, 10.5]
Final	CRY-HOM	7	8	227.1	193.3	33.8	1.72	0.13	0.54	[-1.5, 15.6]
	CRY- WEE	7	5	227.1	160.0	67.1	2.19	0.04	0.74	[0, 16.9]
	HOM-WEE	8	5	193.3	160.0	33.3	0.69	0.73	0.25	[-9.0, 8.0]

Table A4: Post-hoc pairwise comparisons of *Thalassia testudinum* leaf lengths from three systems (CRY: Crystal, HOM: Homosassa, WEE: Weeki Wachee) in the common garden at the initial and final sampling dates. Sample sizes (n), group medians, and median differences between sites are shown. Dunn's test with Bonferroni correction was used for multiple comparisons (Z = standardized test statistic). Effect sizes are quantified using rank-biserial correlation (r) with 95% confidence intervals (CI) calculated from Wilcoxon rank-sum tests.

Sampling date	System pairs (1 vs 2)	n_1	n_2	$Median_1$ (mm)	$Median_2$ (mm)	Median Difference (mm)	Dunn Z	Dunn p-value	r	CI
Initial	CRY-HOM	18	17	195.0	160.0	35.0	1.07	0.43	0.28	[-2.0, 21.0]
	CRY-WEE	18	18	195.0	80.0	115.0	5.08	5.61e-07	0.92	[6.5, 30.0]
	HOM-WEE	17	18	160.0	80.0	80.0	3.94	1.22e-04	0.85	[4.5, 14.0]
Final	CRY- HOM	8	8	157.5	132.5	25.0	0.98	0.49	0.25	[-4.5, 12.0]
	CRY-WEE	8	10	157.5	110.0	47.5	1.64	0.15	0.49	[-1.5, 14.5]
	HOM-WEE	8	10	132.5	110.0	22.5	0.60	0.82	0.14	[-4.0, 8.5]

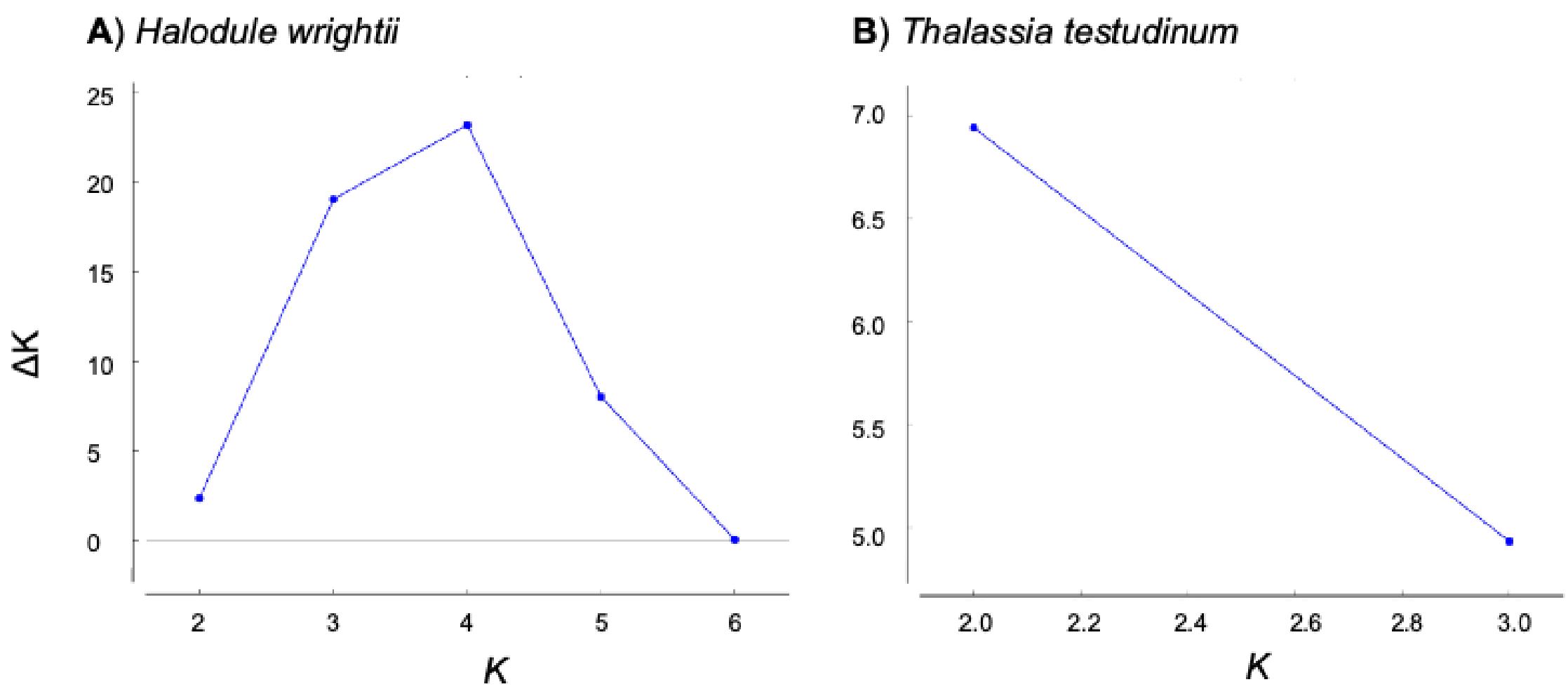

Figure A1: Delta-K plots from STRUCTURE analyses for A) *K* = 2-6 for *Halodule wrightii and* B) *K* = 2-3 for *Thalassia testudinum*.

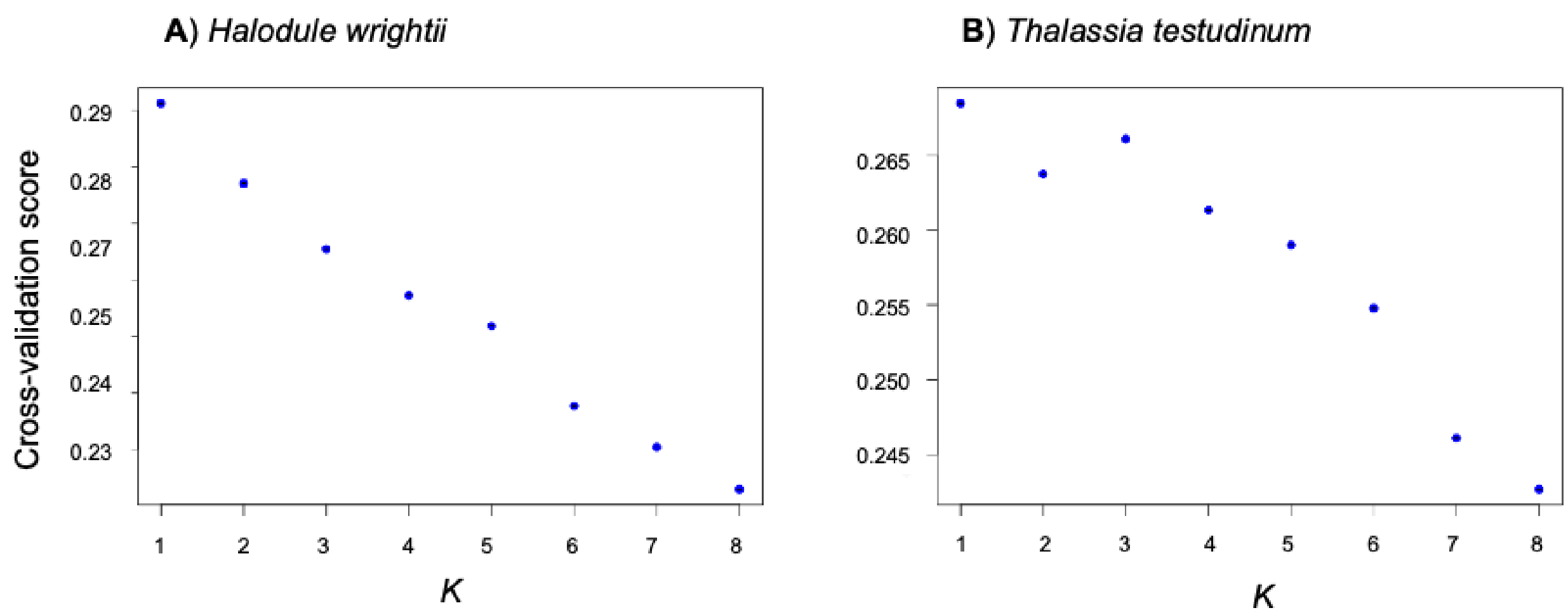

Figure A2: Cross-entropy plots from tess3r analyses for K = 1-8 for A) *Halodule wrightii* and B) *Thalassia testudinum*.
